# Editorial: Orphan crops: breeding and biotechnology for sustainable agriculture, food and nutrition

**DOI:** 10.3389/fpls.2023.1349215

**Published:** 2024-01-04

**Authors:** Zerihun Tadele, Jill M. Farrant, Simon E. Bull, Rita H. Mumm

**Affiliations:** ^1^ Institute of Plant Sciences, University of Bern, Bern, Switzerland; ^2^ Department of Molecular and Cell Biology, University of Cape Town, Cape Town, South Africa; ^3^ Molecular Plant Breeding, Institute of Agricultural Sciences, ETH Zurich, Zurich, Switzerland; ^4^ Plant Biochemistry, Institute of Molecular Plant Biology, ETH Zurich, Zurich, Switzerland; ^5^ African Orphan Crops Consortium, Nairobi, Kenya; ^6^ Department of Crop Sciences, University of Illinois at Urbana-Champaign, Urbana, United States

**Keywords:** orphan crops, underutilized crops, food security, nutrition, biotechnology, crop breeding

Orphan crops are also known as underutilized crops, neglected crops or crops for the future (Tadele, 2019), named for the lack of genetic improvement, not for lack of use or incorporation into cultural diets. These crops play a vital role in the food security and livelihood of resource-poor farmers and consumers particularly in developing countries. Most of these indigenous crops are rich in nutrition (Hunter et al., 2019) and may also be adapted to extreme environmental conditions. Since most fertile land is used to grow primary crops, such as wheat and maize, orphan crops are usually cultivated on less fertile and moisture-deficient soils. As such, orphan crops are expected to be more resilient than primary crops to changing global climates (Mabhaudhi et al., 2019). Despite their significant importance in the present and future agriculture, orphan crops have generally received limited attention by the scientific community, plant breeders, as well as by policy makers.

The ultimate goal of most crop improvement programs is to improve productivity, by raising genetic potential as well as by tackling the major production constraints. Most orphan crops continue to suffer from low productivity, a situation exemplified by comparing the productivity of an orphan crop, millet, and the major crop, wheat, in Asia and Africa over six decades (**Figure 1**). The term “millet” refers to several grain crops grown as staple foods in Asia and Africa. Approximately 15 crops are known as millets, including pearl millet (*Pennisetum glaucum*), finger millet (*Eleusine coracana*), foxtail millet (*Setaria italica*), and proso millet (*Panicum miliaceum*), the most important in a particular region based on the area under cultivation and the amount of production. Although the productivity levels of both wheat and millet were similar in the early 1960s, due to the advancement of research and development of improved varieties of wheat, the grain yield shows substantial increase, while that of millet was not significantly changed from that of the 1960s. It is important to note that the grain yield of wheat grown in Africa has not increased to the level of that in Asia. While wheat productivity has been steadily increasing in Asia, it has fluctuated extensively in Africa due to several reasons, including the environmental conditions and a lack of adapted varieties and required inputs. However, the productivity of wheat in Asia is still low compared to countries in North and West Europe where the grain yield has already reached 10 ton/ha (FAOSTAT, 2023). In 2021, millets were globally cultivated on 31.8 million hectares of land although their total production was only 32.7 million tons (FAOSTAT, 2023).

**Figure 1:**
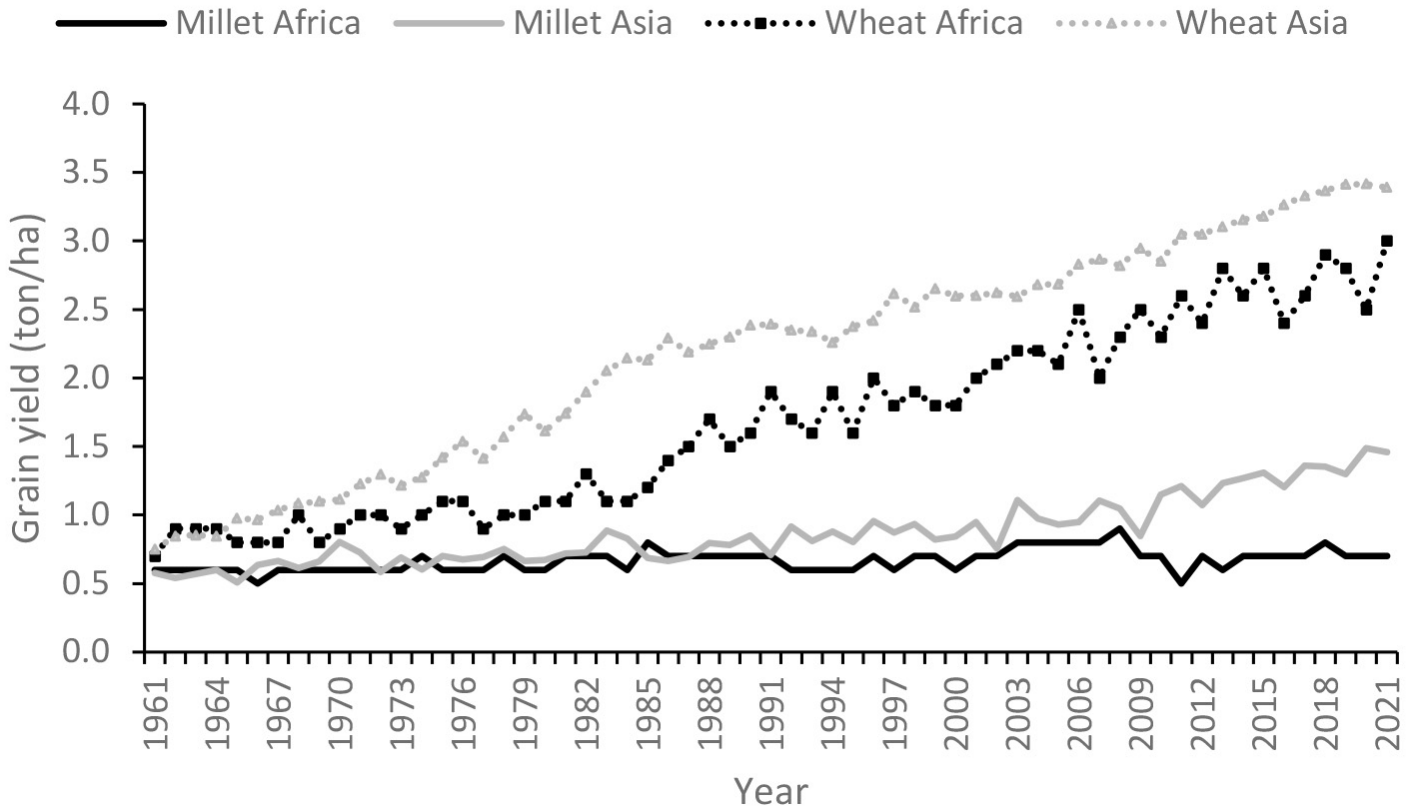
The trend in the grain yield of an orphan crop, millet, and a major crop, wheat, from 1961 to 2021 in Africa and Asia. Millet refers to a group of 15 crops that are extensively cultivated for food and feed in Asia and Africa. Adapted from (FAOSTAT, 2023).

This Research Topic of Frontiers in Plant Science is comprised of 22 articles, of which 17 represent original research and five are review articles. Various categories of orphan crops are featured, including cereals, legumes, vegetables, and root and tuber crops as well as non-food plants. Cereals are represented by finger millet (*Eleusine coracana*) (Anuradha et al.) and pearl millet (*Pennisetum glaucum*) (Pujar et al.) and pseudo-cereals are represented by chia (*Salvia hispanica*) (Gupta et al.), which provides a rich source of protein, fiber and polyunsaturated fatty acids. Pseudo-cereals refer to crops that do not belong to the Grass Family, being dicotyledonous whereas grasses are monocotyledonous. However, pseudo-cereals show their close relationship to the ‘true cereals’ by their nutritional content especially with regards to the carbohydrate composition. Orphan legumes, which are the major protein source, are represented by Bambara groundnut (*Vigna subterranean*) (Olanrewaju et al.), rice bean (*Vigna umbellata*) (Kaul et al.), lablab bean (*Lablab purpureus*) (Letting et al.) and horse gram (*Macrotyloma uniflorum*) (Mahesh et al.). Of importance, Bambara groundnut seeds are highly nutritious and known as a complete food since they contain adequate quantities of protein (19%), carbohydrate (63%), and fat (6.5%) (NRC, 2006). They furthermore have excellent antioxidant properties (Nyau et al., 2015). While lablab bean is native to Eastern Africa, rice bean and horse gram are vital protein sources in Asia (Aditya et al., 2019; Pattanayak et al., 2019). Orphan vegetables are represented by okra (*Abelmoschus esculentus*) (Ibitoye and Kolawole), thickhead or obolo (Crassocephalum crepidiodes*)* (Schramm et al.), and spider plant (*Gynandropsis gynandra*) (Houdegbe et al.). In addition to its use as a leafy vegetable, *C. crepidiodes* is also used as a medicinal plant in Africa. The study published here shows that it accumulates the Pyrrolizidine Alkaloid (PA) Jacobine (Schramm et al.) in response to nitrogen deficiency. Spider plant is rich in vitamins, secondary metabolites and minerals. Sesame (*Sesamum indicum*) (Yol et al.) and shea (*Vitellaria paradoxa*) (Hale et al.) represent oil crops. While sesame is extensively cultivated globally for its high-quality oil and antioxidant lignans, such as sesamin and sesamolin (Moazzami and Kamal-Eldin, 2006), shea is a tree crop grown in the sub-Saharan Africa for both the oil and butter.

Among the root and tuber crops, cassava (*Manihot esculenta*) (Elegba et al.), sweet potato (*Ipomoea batatas*) (Gasura et al.) and enset (*Ensete ventricosum*) (Kidane et al.) are represented. In 2021, cassava was globally cultivated on 30 million hectares of land with the total production of 320 million tons (FAOSTAT, 2023). Cassava is a staple crop for millions of small-holder farmers and consumers particularly in Africa, where its high drought tolerance and outstanding performance on nutrient-poor soils means it survives when other crops would fail. Enset, which is also known as ‘false banana’, is also a staple food for over 20 million people in the densely populated regions of Ethiopia (Borrell et al., 2019).

Robust breeding techniques and statistical tools were reported in several articles. These include the comparison of AMMI and BLUP selection in finger millet (Anuradha et al.); generation mean analysis in pearl millet (Pujar et al.), and breeding potential of Bambara groundnut (Olanrewaju et al.). The tools indicated in the first two papers enable breeders to identify high-yielding genotypes with stable performance, while the review on Bambara groundnut addresses the potential and opportunities of this orphan legume in food and nutritional security, especially in Africa.

Two articles report how to minimize the effect of major pests and diseases, particularly nematodes from the planting material of enset (Kidane et al.) and cassava brown streak disease (CBSD) (Kwibuka et al.). Since nematodes cause severe damage to enset, appropriate management options (Kidane et al.) were suggested to make the planting material free of nematodes. CBSD, which is caused by two virus species, results in up to 100% yield loss in cassava. The authors recommended applying rigorous sanitary systems (Kwibuka et al.) in order to obtain virus-free cassava planting material.

In addition to conventional methods, plant breeding applies genomic technologies such as the use of molecular markers. In this regard, two publications showed how molecular markers are effectively implemented for different purposes. In the first, SNPs were used to map the capsule-shattering trait in sesame (Yol et al.), while in the second, novel ESTs were used in identifying genetic diversity among finger millet accessions (Brhane et al.). Capsule shattering is the major constraint in sesame cultivation, as it hinders mechanized harvesting, and reduces yield. Hence, the discovery of the genes influencing expression of (Yol et al.) capsule shattering and subsequent targeted manipulation could boost sesame productivity substantially. Discovery of novel ESTs has allowed identification of substantial genetic variations (Brhane et al.) for important traits among 55 finger millet landraces.

Whole genome sequencing and/or transcriptomics were reported in four orphan crops. These papers include the *de novo* genome sequence of rice bean (Kaul et al.); genome and transcriptome of horse gram (Mahesh et al.); genomic resources of shea tree (Hale et al.); and a gene expression atlas of chia (Gupta et al.). In addition, the genome sequence of *Pongamia pinnata* (Sreeharsha et al.), a non-food plant with the potential for biodiesel production, was also reported. Information from these whole-genome and/or transcriptome analyses have applications in many areas of plant improvement. These resources set the stage for genomics applications in these crop species.

Three articles focused on biotechnology. The first aims to determine efficient transformation and regeneration protocols for farmer-preferred cassava (Elegba et al.) cultivars, while the remaining two are review papers on the benefits of biotechnology (Zambrano et al.) and perspectives for gene editing (Venezia and Krainer) in orphan crops. Although biotechnology has significant economic benefits to farmers and consumers, there are potential hurdles to commercialization of crop varieties with gene-edited traits in terms of biosafety evaluation and regulatory oversight. Countries such as Kenya and Nigeria lead in terms of biosafety capacity while other African countries are slower to put biosafety infrastructure in place. Gene editing by CRISPR/Cas has excellent potential in improving orphan crops. However, the absence of transformation and regeneration (Venezia and Krainer) methods for most orphan crops may affect the application of the technique in the immediate future.

Two papers focused on the benefits of orphan crops in human nutrition. One describes the leaf elemental composition in spider plant (Houdegbe et al.) while the other reviews aspects of dietary enrichment of orphan crops in marginal environments (Talabi et al.). In the former, a study of 70 spider plant lines revealed significant differences in micronutrient composition, demonstrating the potential to increase nutritive content through plant breeding. In general, zinc, calcium, phosphorus, copper, magnesium, and manganese represented landmark elements (Houdegbe et al.) in the spider plant genotypes.

Several papers collectively investigated the acceptance of new orphan crop varieties by farmers and applied farmers’ selection criteria in their breeding programs. These include: farmers’ feedback on okra characterization (Ibitoye and Kolawole); acceptability of Vitamin-A-biofortified sweet potato (Gasura et al.); and farmers’ participatory selection of lablab bean lines (Letting et al.). The study on okra identified farmers’ preferred traits for establishing breeding programs. In an effort to tackle vitamin-A deficiency in sub-Saharan African countries, 14 newly introduced orange-fleshed sweet potato varieties were investigated for their acceptance by farmers, showing that farmers accepted the dry matter content and taste (Gasura et al.) of all introduced varieties. Based on a study with 31 farmers in five districts in Tanzania, farmers’ preferred traits (Letting et al.) were identified in lablab bean, with pest and disease resistances, early maturity, and high yield prioritized as the most important traits.

Lastly, the review paper on the exploitation of orphan legumes for food, income, and nutrition security (Popoola et al.) provides an excellent overview on the potential of African crops for improving the nutrition of the African population, and points to the need for genetic improvement of these highly nutritious, culturally-important crops using modern breeding and genomic techniques.

In general, the papers in this Research Topic provide substantial information to the crop improvement community and policy makers. It is important to provide due attention to the need for more productive varieties of orphan crops (Tadele, 2019) since these neglected crops in terms of scientific research deserve modern improvement approaches such as speed breeding (Chiurugwi et al., 2019), advanced molecular breeding techniques (Ribaut and Ragot, 2019), and applications of genomics (Jamnadass et al., 2020). In addition, the benefits of orphan crops in human nutrition and health need to be promoted by relevant stakeholders to advance their cultivation and further expand their consumption (Knez et al., 2023).

## Author contributions

ZT: Writing – original draft, Writing – review & editing. JF: Writing – original draft, Writing – review & editing. SB: Writing – original draft, Writing – review & editing. RM: Writing – original draft, Writing – review & editing.
